# Real-World Treatment Patterns and Persistence of Omidenepag Isopropyl in Korean Patients with Normal-Tension Glaucoma

**DOI:** 10.3390/jcm15208200

**Published:** 2026-10-21

**Authors:** Na Hee Kim, Youn Hye Jo

**Affiliations:** Department of Ophthalmology, Konkuk University Medical Center, Konkuk University School of Medicine, Seoul 05030, Republic of Korea

**Keywords:** omidenepag isopropyl, normal-tension glaucoma, treatment persistence, prostaglandin-associated periorbitopathy, EP2 receptor agonist, real-world evidence

## Abstract

**Background/Objectives:** Prostaglandin analogues may cause prostaglandin-associated periorbitopathy (PAP). Omidenepag isopropyl (OMDI), a selective EP2 receptor agonist, may provide an alternative, but real-world data in normal-tension glaucoma (NTG) are limited. We evaluated treatment patterns, persistence, and intraocular pressure (IOP) outcomes of OMDI in Korean patients with NTG. **Methods:** This retrospective study included 403 eyes of 218 patients with NTG who initiated OMDI and were followed for up to 12 months. Patients received monotherapy, adjunctive therapy, or switching therapy. IOP was analyzed using a three-level linear mixed-effects model, and persistence was assessed using Kaplan–Meier analysis. **Results:** OMDI was prescribed as monotherapy in 88 patients (40.4%), adjunctive therapy in 25 (11.5%), and switching therapy in 105 (48.2%). Among switchers, PAP was the most common reason (68.6%). IOP decreased significantly in all groups (all *p* < 0.001), with model-estimated 12-month changes of −4.08, −3.37, and −1.40 mmHg, respectively. Twelve-month persistence was 79.8%. OMDI was discontinued in 44 patients (20.2%), most commonly because of visual disturbance or decreased vision (38.6%). **Conclusions:** OMDI provided sustained IOP reduction and 79.8% 12-month persistence in Korean patients with NTG and was frequently selected as an alternative to prostaglandin analogues in patients with PAP.

## 1. Introduction

Glaucoma is a chronic, progressive optic neuropathy characterized by structural changes in the optic disc and retinal nerve fiber layer that lead to progressive retinal ganglion cell death and corresponding visual field impairment [1,2]. Among topical medications, prostaglandin analogues are generally recommended as first-choice therapy. They lower intraocular pressure (IOP) potently, cause few systemic side effects, and are dosed once daily, which supports adherence [3]. However, prolonged stimulation of the prostanoid FP receptor is associated with prostaglandin-associated periorbitopathy (PAP), which includes deepening of the upper eyelid sulcus, periorbital fat atrophy, and eyelid tightening. These periocular changes may be cosmetically undesirable to patients and have also been associated with less favorable outcomes after glaucoma filtration surgery [4,5,6].

Omidenepag isopropyl (OMDI) 0.002% (EYBELIS ophthalmic solution 0.002%, Santen Pharmaceutical Co., Ltd., Osaka, Japan) was developed as a selective E-prostanoid subtype 2 (EP2) receptor agonist [7,8,9]. By targeting EP2 receptors in the trabecular meshwork and ciliary body, OMDI enhances both trabecular and uveoscleral outflow without activating FP receptors. In the AYAME phase 3 clinical study, OMDI demonstrated non-inferiority to latanoprost 0.005% in lowering IOP among patients with ocular hypertension (OHT) or primary open-angle glaucoma (POAG) [10]. Subsequent studies have also confirmed its efficacy in patients with normal-tension glaucoma (NTG) [11].

Several previous real-world studies have evaluated the efficacy, safety, and treatment persistence of OMDI in Japanese populations with heterogeneous glaucoma subtypes [12,13]. Evidence confined to NTG remains limited, however, and little has been reported on the clinical circumstances in which OMDI is selected in this population or on how long patients remain on it. We therefore examined the real-world use of OMDI in Korean patients with NTG, taking 12-month treatment persistence as the primary outcome.

## 2. Methods

This retrospective observational study included consecutive patients NTG who initiated treatment with omidenepag isopropyl 0.002% (OMDI; Eybelis^®^, Santen Pharmaceutical Co., Osaka, Japan) at Konkuk University Medical Center between 1 October 2021 and 30 October 2022. Patients were followed for up to 12 months after OMDI initiation. The study protocol was reviewed by the Institutional Review Board of Konkuk University Medical Center and was determined to be exempt from ethical review. The requirement for informed consent was waived because of the retrospective nature of the study and the use of de-identified clinical data. The study was conducted in accordance with the tenets of the Declaration of Helsinki.

NTG was defined as glaucomatous changes to the optic nerve accompanied by corresponding visual field defects, confirmed through at least two consecutive examinations. Patients included in the study had open angles with documented untreated IOP measurements below 21 mmHg [14]. For the present analyses, baseline IOP was defined as the IOP measured immediately before OMDI initiation. Pseudophakic or aphakic eyes were excluded because of the reported risk of cystoid macular edema associated with OMDI [15]. OMDI was not administered concomitantly with prostaglandin analogues. Eyes with a history of retinal diseases or uveitis were excluded from the study.

Patients were classified into three treatment groups according to the clinical indication for OMDI: (1) monotherapy, in which OMDI was prescribed as the initial glaucoma medication; (2) adjunctive therapy, in which OMDI was added to existing glaucoma medications to achieve additional IOP reduction; and (3) switching therapy, in which OMDI replaced a previous topical glaucoma medication. In bilateral cases, both eyes were treated using the same OMDI treatment strategy. For patients receiving switching therapy, the primary reason for switching to OMDI was determined from the medical record and categorized as prostaglandin-associated periorbitopathy (PAP), insufficient IOP-lowering efficacy or glaucoma progression, other adverse effects, dissatisfaction with instillation frequency, change to monocular treatment, inadequate response to a non-prostaglandin medication, or other clinical considerations. PAP was identified retrospectively from routine clinical documentation based on physician-documented periocular changes and/or patient-reported cosmetic changes during prostaglandin analogue treatment. Clinical photographs were available for some patients but were not systematically obtained before and after switching in all patients; therefore, PAP classification was based primarily on clinical documentation rather than standardized photographic assessment.

Baseline demographic and ocular characteristics, including age, sex, baseline IOP, central corneal thickness (CCT), axial length, previous glaucoma medications, and treatment strategy, were obtained from electronic medical records. IOP was measured using a Goldmann applanation tonometer (AT 900; Haag-Streit AG, Köniz, Switzerland) at baseline and 1, 6, and 12 months after OMDI initiation, and CCT was measured using ultrasound pachymetry (AL-2000; Tomey Corporation, Nagoya, Japan). Axial length was not routinely measured in all patients and was therefore available only for a subset of eyes.

The primary outcome was treatment persistence at 12 months. Secondary outcomes were the distribution of treatment strategies, the documented reason for switching, longitudinal IOP change, and the documented reason for discontinuation. Treatment persistence was defined as continued use of OMDI throughout the observation period. Treatment discontinuation was defined as permanent cessation of OMDI documented in the medical record for any reason. For patient-level analyses, a discontinuation event was defined as permanent cessation of OMDI in all treated eyes. No patient receiving bilateral OMDI discontinued treatment in only one eye while continuing treatment in the fellow eye. Reasons for discontinuation were categorized as visual disturbance, ocular pain, conjunctival hyperemia, cystoid macular edema, insufficient IOP control, stinging or irritation, or other causes.

### Statistical Analysis

Continuous variables are presented as mean ± standard deviation (SD), and categorical variables as frequencies and percentages. Patient-level continuous variables were compared using one-way analysis of variance, whereas eye-level continuous variables were compared using linear mixed-effects models with a patient-level random intercept to account for inter-eye correlation. Categorical variables were compared using the chi-square test or Fisher’s exact test, as appropriate.

Longitudinal IOP outcomes were analyzed using a three-level linear mixed-effects model with fixed effects for treatment group, follow-up time, and their interaction, and random intercepts for patients and eyes nested within patients to account for correlation between fellow eyes and repeated measurements within the same eye. IOP measurements obtained after permanent discontinuation of OMDI were excluded from the longitudinal IOP analysis. Variance components were estimated using restricted maximum likelihood. Intraclass correlation coefficients were calculated from the estimated variance components. Treatment persistence was estimated using the Kaplan–Meier method, with discontinuation of OMDI defined as the event. Treatment status was ascertained from the medical records. Patients with documented continued OMDI use at the 12-month follow-up were administratively censored at 12 months.

To identify factors associated with treatment discontinuation because of visual disturbance, univariable logistic regression analyses were performed using age, sex, history of laser-assisted in situ keratomileusis (LASIK) or laser-assisted subepithelial keratectomy (LASEK), baseline IOP, and treatment strategy as candidate predictors. For patient-level logistic regression, baseline IOP was calculated as the arithmetic mean of the baseline IOP measurements of both treated eyes in bilateral cases and as the IOP of the treated eye in unilateral cases. Given that only 17 visual disturbance-related discontinuation events occurred, a multivariable model including all candidate predictors would require six regression coefficients and was considered at high risk of overfitting. Analyses of potential predictors were therefore considered exploratory and limited to univariable logistic regression. Odds ratios (ORs) with 95% confidence intervals (CIs) were calculated. All statistical analyses were performed using Python version 3.13 (Python Software Foundation, Wilmington, DE, USA) with the statsmodels and lifelines packages. A two-sided *p* value < 0.05 was considered statistically significant.

## 3. Results

### 3.1. Patient Characteristics

A total of 218 patients (403 eyes) who initiated omidenepag isopropyl (OMDI) treatment were included in this study. Of the 218 patients, 185 contributed both eyes and 33 contributed one eye. OMDI was prescribed as monotherapy in 88 patients (40.4%), as adjunctive therapy in 25 (11.5%), and as switching therapy in 105 (48.2%), according to the clinical indication at initiation. IOP measurements were available from 218 patients (403 eyes) at baseline, 218 patients (403 eyes) at 1 month, 184 patients (338 eyes) at 6 months, and 174 patients (318 eyes) at 12 months, yielding a total of 1462 eye-level IOP observations. Baseline demographic and ocular characteristics are summarized in Table 1.

Age and sex distributions were comparable among the three groups. Baseline IOP differed significantly among treatment groups (*p* < 0.001), with the highest baseline IOP observed in the monotherapy group. Other ocular characteristics, including central corneal thickness and axial length, did not differ significantly among treatment groups.

### 3.2. Reasons for Switching to OMDI

Among the 105 patients in the switching group, PAP was the most common reason for switching to OMDI (72 patients, 68.6%), followed by insufficient IOP-lowering efficacy or glaucoma progression (18, 17.1%) and other adverse effects (9, 8.6%). Less common reasons included dissatisfaction with instillation frequency (3, 2.9%), change to monocular treatment (2, 1.9%), and inadequate response to a non-prostaglandin medication (1, 1.0%).

### 3.3. Longitudinal IOP Outcomes

**Figure 1:**
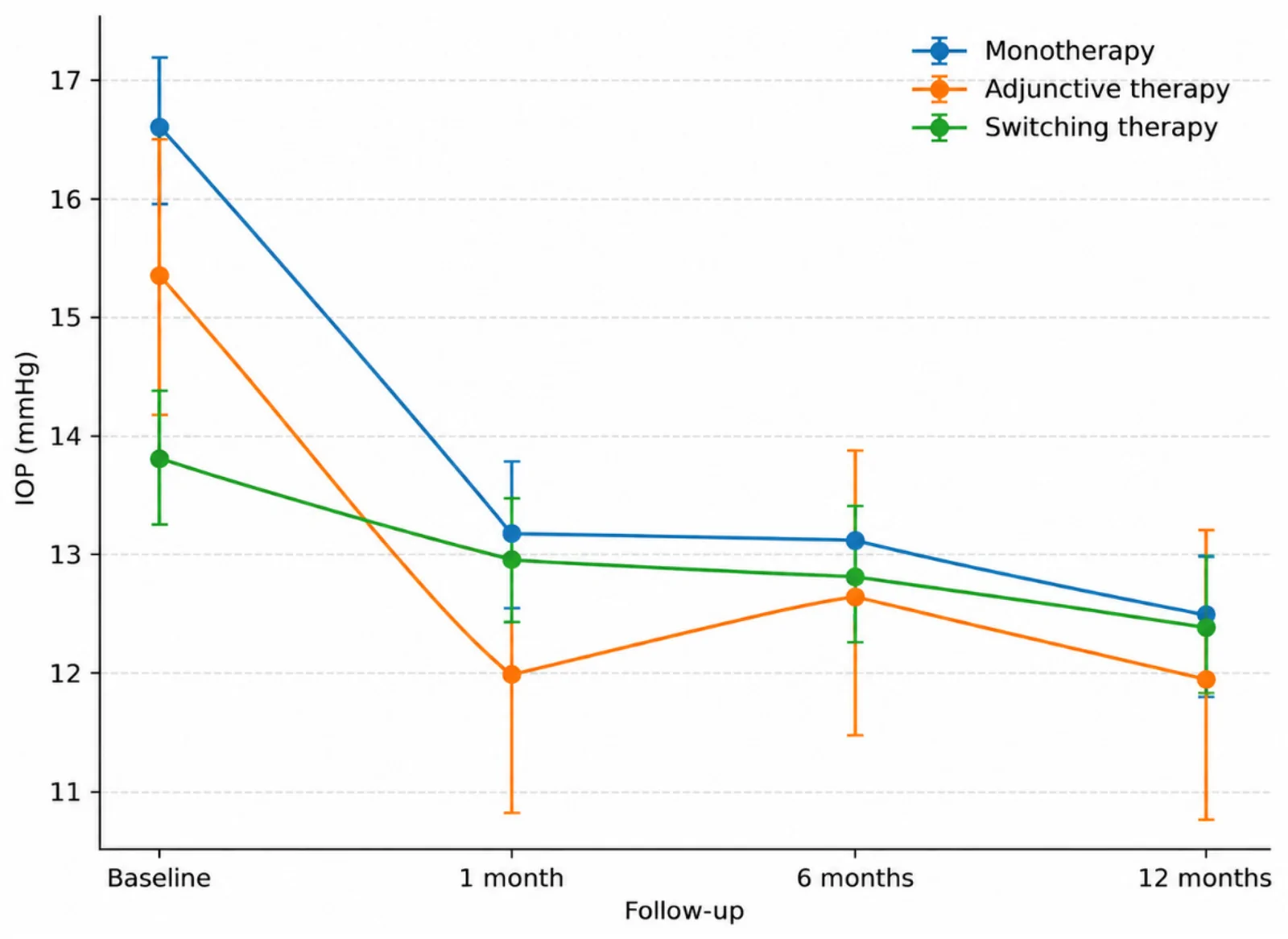
Longitudinal changes in intraocular pressure after initiation of omidenepag isopropyl according to treatment strategy.

Longitudinal IOP outcomes are summarized in Figure 1. Linear mixed-effects modeling demonstrated significant reductions in IOP following OMDI initiation in all treatment groups (all *p* < 0.001). Estimated mean IOP remained significantly lower than baseline throughout the 12-month follow-up period.

Estimated mean intraocular pressure (IOP) at baseline and at 1, 6, and 12 months after initiation of omidenepag isopropyl (OMDI) is shown for the monotherapy, adjunctive therapy, and switching therapy groups. Error bars represent 95% confidence intervals derived from the linear mixed-effects model. IOP decreased significantly from baseline in all three treatment groups during follow-up (all *p* < 0.001).

The greatest absolute reduction was observed in the monotherapy group, which had the highest baseline IOP. However, sustained IOP lowering was observed in all three treatment groups. At 12 months, the estimated mean IOP was 12.49 mmHg in the monotherapy group, 11.96 mmHg in the adjunctive therapy group, and 12.39 mmHg in the switching therapy group, corresponding to model-estimated changes from baseline of −4.08, −3.37, and −1.40 mmHg, respectively.

The between-patient variance was 6.31, the residual variance was 4.08, and the additional variance attributable to eyes nested within patients was negligible. The patient-level The intraclass correlation coefficient (ICC) was 0.61.

### 3.4. Treatment Persistence and Reasons for Discontinuation

**Figure 2:**
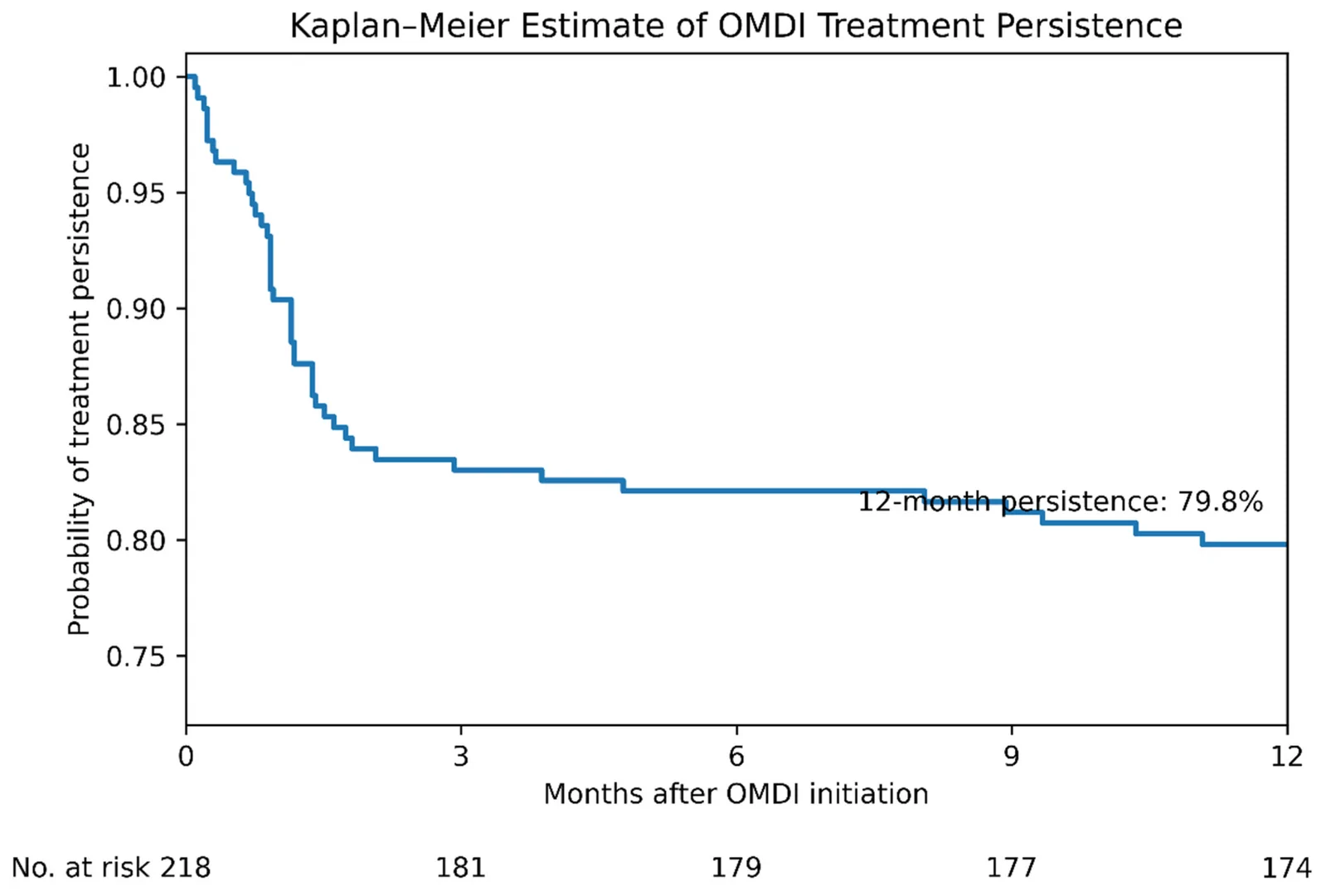
Kaplan–Meier estimate of treatment persistence with omidenepag isopropyl.

Overall, 44 of 218 patients (20.2%) discontinued OMDI during the observation period. Continued OMDI use at the 12-month follow-up was confirmed from the medical records for all 174 patients who had not discontinued treatment. The Kaplan–Meier estimated probability of treatment persistence at 12 months was 79.8%. The numbers at risk at baseline and at 3, 6, 9, and 12 months were 218, 181, 179, 177, and 174, respectively (Figure 2).

Kaplan–Meier curve showing the probability of remaining on omidenepag isopropyl (OMDI) during the 12-month follow-up period. The estimated probability of treatment persistence at 12 months was 79.8%. Numbers at risk are shown below the graph.

Visual disturbance or decreased vision was the most common reason for discontinuation, occurring in 17 patients (38.6% of discontinuers), followed by ocular pain and conjunctival hyperemia in 9 patients each (20.5%). Other reasons accounted for 4 discontinuations (9.1%), and insufficient IOP control and stinging or irritation each accounted for 2 (4.5%). The reason was not documented in 1 patient (2.3%). No cases of cystoid macular edema were documented during the 12-month follow-up period. Reasons for discontinuation are summarized in Table 2.

A review of the 17 patients who discontinued OMDI because of visual disturbance or decreased vision showed that the reported symptoms included blurred or hazy vision, decreased vision, glare, and difficulty with near vision. Among patients for whom the timing of symptom onset was documented, several reported symptoms within 1–2 weeks after treatment initiation. Paired visual acuity and autorefraction measurements before and after symptom onset were not consistently available, as these examinations were not systematically performed in this retrospective study and many patients had already discontinued OMDI before their scheduled follow-up visit. Objective myopic shifts of approximately 1.0 D and 1.25 D were documented in two patients. In both patients, visual acuity and refraction had fully returned to baseline at the 1-month follow-up after OMDI discontinuation. Several other patients also reported subjective improvement after discontinuation.

The table shows the reasons for treatment discontinuation among the 44 patients who discontinued omidenepag isopropyl (OMDI) during follow-up. Percentages were calculated using the number of patients who discontinued treatment as the denominator. Visual disturbance or decreased vision was the most common reason for discontinuation, accounting for 38.6% of discontinuations.

### 3.5. Predictors of Visual Disturbance-Related Discontinuation

Univariable logistic regression analyses were performed to identify factors associated with discontinuation due to visual disturbance (Table 3). None of the evaluated variables was significantly associated with visual disturbance-related discontinuation. Baseline IOP showed the strongest association but did not reach statistical significance (OR, 0.90 per 1-mmHg increase; 95% CI, 0.78–1.03; *p* = 0.134). A history of LASIK/LASEK was associated with an OR of 2.18, although the confidence interval was wide (95% CI, 0.57–8.30; *p* = 0.254). Age, sex, and treatment strategy were not significantly associated with discontinuation.

## 4. Discussion

In this real-world cohort of Korean patients with normal-tension glaucoma, OMDI was used both to lower IOP and as an alternative in patients who developed PAP during long-term prostaglandin analogue therapy. Previous studies have demonstrated the IOP-lowering efficacy and safety of OMDI [10,11,12], but data on how OMDI is used specifically in patients with NTG are limited.

Roughly two thirds of switches were prompted by PAP, whereas inadequate pressure control or documented progression accounted for fewer than one in five. Periocular appearance was therefore the dominant driver of medication change in a population whose IOP was already within the statistically normal range. This aligns with the pharmacology: OMDI acts at the EP2 receptor and does not engage the FP receptor implicated in inhibition of adipogenesis and orbital fat atrophy [7,8,9]. Nakakura et al. and Sakata et al. each documented alleviation of periorbital changes after substitution of an FP receptor agonist with OMDI [16,17]; in our cohort, this was the principal reason for choosing OMDI.

Four of five patients remained on OMDI at 12 months. Nakakura et al. reported a 12-month Kaplan–Meier persistence of 70% in a Japanese two-institute cohort [18]. The crude proportion of patients meeting the respective failure criterion was nonetheless almost identical (20.6% versus 20.2% in our cohort); the difference between the Kaplan–Meier estimates may partly reflect differences in follow-up duration and censoring. The two definitions also differ in one respect: that study counted the addition of a second agent to ongoing OMDI as a failure, whereas we counted only permanent cessation of OMDI, including cessation prompted by inadequate pressure control.

Discontinuation was driven by ocular symptoms. Visual disturbance or decreased vision was the leading reason for discontinuation, accounting for nearly as many discontinuations as conjunctival hyperemia and ocular pain combined, whereas only two patients stopped because pressure control proved inadequate. Transient myopic shift and blurring after OMDI initiation have been reported [19,20]. The mechanism of OMDI-associated myopic shift remains uncertain. Previous studies have reported transient changes in corneal curvature and central corneal thickness after OMDI initiation [19,20], while a recent prospective study also found increased lens thickness and decreased anterior chamber depth [19]. These changes may alter corneal refractive power and lens position or accommodation and contribute to the transient myopic shift. Ciliary muscle spasm has also been proposed as a possible mechanism [19]. Consistent with previous reports, objective myopic shifts were documented in two patients in our cohort, and both visual acuity and refraction returned to baseline at the 1-month follow-up after OMDI discontinuation. However, most visual complaints were subjective, and paired refractive measurements were not consistently available. Therefore, the extent to which the visual disturbances observed in our cohort were attributable to OMDI-induced refractive changes could not be determined. Even transient changes in visual quality may interfere with daily activities, and the possibility of such symptoms should be discussed before treatment initiation.

IOP remained below baseline in every group throughout the 12 months, and the estimated means converged near 12 mmHg irrespective of how OMDI was introduced. A recent Korean prospective multicenter phase IV study by Bae et al. provides complementary local context, reporting a 16% mean IOP reduction at 12 weeks in a treatment-naïve NTG subgroup (n = 31) [21]. Although its prospective design and shorter follow-up differ from those of the present study, the findings provide additional Korean data on the IOP-lowering effect of OMDI in NTG. Inoue et al. reported comparable one- and three-year reductions in Japanese patients with NTG [22,23]. The absolute change was largest in the monotherapy group and smallest in the switching group, but these values are not directly comparable. Baseline IOP in the monotherapy group represented an untreated pressure, whereas baseline IOP in the switching group represented a pressure already lowered by the medication that OMDI replaced. Thus, the smaller model-estimated reduction in the switching group (−1.40 mmHg) reflects its already-treated baseline and should not be interpreted as reduced IOP-lowering efficacy of OMDI.

No baseline characteristic was significantly associated with discontinuation because of visual disturbance. Although the point estimate for previous LASIK/LASEK suggested higher odds of discontinuation, the confidence interval was wide (OR, 2.18; 95% CI, 0.57–8.30) and the association was not statistically significant. Given the small number of visual disturbance-related discontinuation events, the wide confidence intervals indicate substantial uncertainty in these exploratory estimates, and the point estimates should therefore be interpreted cautiously. Whether previous corneal refractive surgery modifies susceptibility to OMDI-associated myopic shift remains uncertain. Because objectively documented myopic shift occurred in only two patients, our data cannot address this question directly. The limited number of events precluded reliable multivariable adjustment; therefore, these univariable associations should not be interpreted as independent predictors, and residual confounding cannot be excluded.

This study has several limitations. First, this was a retrospective study performed at a single tertiary referral center, and the exclusion of pseudophakic and aphakic eyes further limits the generalizability of the findings to the broader glaucoma population. Second, treatment initiation and discontinuation dates were obtained retrospectively from chart review, and the date of the last recorded prescription may postdate actual cessation; persistence estimates may therefore be modestly optimistic. Persistence is also sensitive to how failure is defined—we counted only permanent cessation, not therapy changes—and we lacked a concurrent comparator arm on an FP receptor agonist. Third, the number of discontinuations due to visual disturbance was small, limiting the statistical power of the exploratory logistic regression analyses. Fourth, visual acuity and refractive measurements were not systematically obtained at the onset of visual symptoms because of the retrospective nature of the study, and many patients had already discontinued OMDI before their scheduled follow-up visit. Therefore, the mechanism underlying visual disturbance could not be objectively determined in most patients. Fifth, adverse events that did not result in treatment discontinuation may not have been systematically captured in the medical records; therefore, the present study should not be interpreted as a comprehensive safety evaluation of OMDI. Sixth, IOP measurements were obtained during routine clinical visits and were not standardized by time of day; therefore, diurnal variation may have contributed to variability in the observed IOP changes. Finally, treatment groups were defined according to clinical treatment strategy rather than random assignment, and baseline IOP differed among groups; therefore, direct comparisons of the magnitude of IOP reduction among treatment groups should be interpreted cautiously.

## 5. Conclusions

In this Korean NTG cohort, OMDI was frequently selected as an alternative in patients with prostaglandin-associated periorbitopathy and was associated with sustained IOP reduction and 79.8% treatment persistence at 12 months. Visual disturbance or decreased vision was the most frequently documented reason for discontinuation.

## Figures and Tables

**Table 1:** Baseline demographic and ocular characteristics.

Characteristic	Overall (218/403)	Monotherapy (88/154)	Adjunctive (25/43)	Switching (105/206)	*p* Value
**Patient-level variables**					
Age, years	53.50 ± 12.08	54.10 ± 12.04	49.72 ± 15.80	53.90 ± 11.04	0.250
Female, n (%)	120 (55.0)	45 (51.1)	13 (52.0)	62 (59.0)	0.518
**Eye-level variables**					
Baseline IOP, mmHg	15.02 ± 4.16	16.65 ± 3.56	15.12 ± 5.41	13.78 ± 3.86	<0.001
CCT, μm	548.26 ± 44.67	549.19 ± 45.91	546.26 ± 40.79	547.98 ± 44.70	0.877
Axial length, OD, mm	25.55 ± 1.94 (n = 101)	25.14 ± 1.61 (n = 56)	26.36 ± 2.78 (n = 12)	25.94 ± 1.97 (n = 33)	0.051
Axial length, OS, mm	25.47 ± 1.81 (n = 102)	25.16 ± 1.59 (n = 58)	26.34 ± 2.65 (n = 11)	25.73 ± 1.78 (n = 33)	0.084

Values are mean ± SD or n (%). Age and sex are patient-level variables. Baseline IOP and CCT are eye-level variables; group comparisons account for clustering within patients. Axial length is presented separately for OD and OS with the available sample size. IOP, intraocular pressure; CCT, central corneal thickness; OD, right eye; OS, left eye.

**Table 2:** Treatment discontinuation and reasons.

**Measure**	**n**	**Denominator**	**Percent**
Patients discontinuing OMDI	44	218	20.2%
Eyes discontinuing OMDI	85	403	21.1%
**Reason (patient level)**	**n**	**Denominator**	**Percent**
Visual disturbance/decreased vision	17	44	38.6%
Ocular pain	9	44	20.5%
Conjunctival hyperemia	9	44	20.5%
Other	4	44	9.1%
Insufficient IOP control	2	44	4.5%
Stinging/irritation	2	44	4.5%
Unclassified	1	44	2.3%

OMDI, omidenepag isopropyl; IOP, intraocular pressure; n, number.

**Table 3:** Univariable logistic regression for visual disturbance-related discontinuation.

Predictor	N	Events	OR	95% CI Lower	95% CI Upper	*p* Value
Age, per 1 year	218	17	1.010	0.968	1.053	0.653
Female sex	218	17	1.547	0.551	4.346	0.407
History of LASIK/LASEK	218	17	2.179	0.572	8.301	0.254
Baseline IOP, per 1 mmHg	218	17	0.898	0.780	1.034	0.134
Treatment: Adjunctive vs. Monotherapy	218	17	0.417	0.050	3.500	0.420
Treatment: Switching vs. Monotherapy	218	17	0.825	0.296	2.296	0.712
Treatment group (global test)	218	17	N/A	N/A	N/A	0.670

OR, odds ratio; CI, confidence interval; IOP, intraocular pressure; LASIK, laser-assisted in situ keratomileusis; LASEK, laser-assisted subepithelial keratectomy.

## Data Availability

The data presented in this study are available on reasonable request from the corresponding author. The data are not publicly available due to privacy and ethical restrictions concerning patient information.
